# The Behavioral and Mental Health Benefits of Speaking the Heritage Language within Immigrant Families: The Moderating Role of Family Relations

**DOI:** 10.1007/s10964-023-01807-5

**Published:** 2023-06-24

**Authors:** Elina Kilpi-Jakonen, Hye Won Kwon

**Affiliations:** INVEST Research Flagship Centre, University of Turku, Turku, Finland

**Keywords:** Heritage language, Externalizing problems, Internalizing problems, Family relations, Children of immigrants, CILS4EU

## Abstract

Understanding the development of behavioral and mental health issues among adolescents, particularly those from immigrant families, is a key area of concern. Many prior studies have focused on the role of societal (country-of-destination) language skills, but we know less about the role played by the use of the heritage language in families. We examined this latter relationship with a focus on changes in heritage language use and internalizing and externalizing problems, and how family relations moderate this relationship. We used the first two waves (2010/2011 and 2011/2012) of the Children of Immigrants Longitudinal Survey in Four European Countries (CILS4EU) data collected from Germany (*n* = 1614; M_age_ = 14.8 years, 50% female), the Netherlands (*n* = 1203; M_age_ = 14.7 years, 54% female), Sweden (*n* = 1794; M_age_ = 14.2 years, 53% female), and England (*n* = 1359; M_age_ = 14.6 years, 50% female). Our results suggest that increased use of heritage language is associated with fewer externalizing problems only in families with greater family cohesion and parental warmth (in Germany and the U.K.) and with fewer internalizing problems only in families with higher parental monitoring (in the Netherlands and Sweden). Good family relations are thus an important precondition for increased heritage language use to lead to improved behavioral and mental health for children of immigrants.

## Introduction

How behavioral and mental health develop over adolescence is a key issue of concern. A group of particular importance are children from immigrant families who may face additional challenges in comparison to their majority peers as they deal with identity development in relation to both the surrounding society as well as their ethnic heritage (e.g., Mood et al., 2016). Language is often a key aspect of ethnic heritage as well as being highly linked to (ethnic) social relationships both within and outside the family (e.g., Wang et al., 2022), yet its specific role for behavioral and mental health has not been widely studied, particularly from a longitudinal perspective. Furthermore, little is known about whether the role of heritage language use is conditional on family relations: increased use of the heritage language may be beneficial in family settings characterized by warmth and cohesiveness whereas reduced use of the heritage language may even alleviate adolescents’ problems when the family is not supportive. Thus, in a longitudinal framework, this study examines the moderating role of family relations in the association between changes in heritage language use and changes in behavioral and mental health with nationally representative samples of children of immigrants in four European countries: Germany, the Netherlands, Sweden, and the U.K.

### Immigrant Children’s Well-Being and the Use of Heritage Language within Immigrant Families

A great deal of literature has studied the behavioral and mental health of children of immigrants (e.g., Mood et al., 2016, Delaruelle et al., 2021). One of the widely-studied factors contributing to better adaptation outcomes is language use and proficiency. It is fairly well established that children of immigrants benefit from learning the societal (host) language (i.e., there is a cost of limited proficiency of the societal language), particularly in terms of sociocultural adaptation but also for psychological adaptation. For example, limited English proficiency has been linked to worse behavioral and mental health among both Latino and Asian American children (Dawson & Williams, 2008; Kang et al., 2014), whereas lower German proficiency is associated with lower subjective well-being among Turkish-origin youngsters in Germany (Koydemir, 2013).

However, whether using the heritage language is beneficial for better adaptation of immigrant children has been debated among scholars particularly from three theoretical frameworks (see Zhou 1997 for a review). Traditional assimilation theory argues that immigrant children who use their heritage language are less likely to develop proficiency in the societal language which, in turn, negatively affects their adaptation. By contrast, segmented assimilation theory argues that maintaining the heritage language is beneficial for immigrant children’s adaptation (e.g., Bankston & Zhou, 1995; Feliciano, 2001). This is partly because the heritage language is not only a means of communication within families but also a way of connecting to the wider ethnic community. This also links heritage language use to different acculturation strategies, whereby it has been argued that identification with and cultural attachment to both the mainstream society as well as the co-ethnic group is more beneficial for children of immigrants than only turning toward one (or neither) of these (e.g., Berry, 1997; Berry & Sam, 1996). Heritage language use is thus also part of the identity formation process (e.g., Leyendecker et al., 2018; Oh & Fuligni, 2010; Wang et al., 2022) that may further relate to the behavioral and mental health of immigrant children. Ethnic identity and stronger connectedness to ethnic communities, which is partly reliant on heritage language fluency, protect immigrant children from unhealthy behaviors and help them sustain better mental well-being (Luthra et al., 2020; Yoon et al., 2008). It has also been proposed that parental heritage language use promotes children’s emotional development better than switching to the societal language, and this promotes behavioral and mental health (Chen et al., 2012).

Prior studies have largely focused on heritage language proficiency, rather than the heritage language use per se, for adjustment outcomes, and produced rather inconsistent findings across studies and populations: some report behavioral and mental health benefits of heritage language proficiency (e.g., Chung et al., 2019; Collins et al., 2011; Wang et al., 2022; foreign-born Chinese American youth in Liu et al., 2009) while others show no association between heritage language proficiency and mental health (e.g., the U.S.-born Chinese American immigrant youth in Liu et al. 2009) or even a negative association between the two (Ren et al., 2016). A meta-analysis published approximately one decade ago did not find evidence to suggest that proficiency in the heritage language would be associated with mental health outcomes (Yoon et al., 2013).

Compared to the past scholarship on societal and heritage language proficiency, there is a surprising dearth of research on the heritage language use in immigrant families (see also De Houwer, 2017). Yet continued use of the heritage language at home is of primary importance for children’s heritage language skills to develop (Chumak-Horbatsch & Garg, 2006; De Houwer, 2007; Tran, 2010), and adolescence is likely to be a critical period for the continued use of heritage language since both the education system and the peer group may place their own pressures on increasing use of the societal language. Moreover, there is a need to study the longitudinal implications of heritage language use for adolescent behavioral and mental health (e.g., Kunst, 2021; Müller et al., 2020). A review of the association between heritage language maintenance and well-being (where well-being includes a variety of measures including family relations and depressive symptoms) concluded that the evidence suggests that there is an association between the two, though highlighting that the directionality of this link needs to be further analyzed (Müller et al., 2020). Indeed, longitudinal analyses of the adaptation of children of immigrants have been rather rare and the issue of a “causality crisis” in this regard has been raised (Kunst, 2021). Nevertheless, some longitudinal analysis exists. For example, among recently-immigrated Latino youth the loss of the heritage culture, where the measure included language use, was identified as a risk for behavioral and mental health problems (Meca et al., 2018). Another longitudinal study found that among Asian Americans followed from kindergarten to fifth grade, the lowest rate of growth in problem behaviors was among those who use their heritage language at home but are also proficient in English at school entry (Han & Huang, 2010).

### Family Relations as an Important Moderator

Language use is also closely linked to family bonding and children’s relationships with their parents (Leyendecker et al., 2018; Müller et al., 2020). Prior research has documented the importance of the family environment in determining the behavioral and mental health outcomes of adolescents with immigrant backgrounds (e.g., Delaruelle et al., 2021; Harker, 2001; Montazer & Wheaton, 2011; Mood et al., 2017). Linking heritage language use, family relations, and behavioral and mental health problems of adolescents, past studies have examined whether family environment mediates the relationship between heritage language proficiency and behavioral and mental health. When language proficiency has been studied as part of ethnic acculturation (or enculturation), family relations have been found to mediate the relationship between (strong) ethnic acculturation and (better) mental health outcomes among immigrant adolescents from the former Soviet Union in the U.S. (Birman & Taylor-Ritzler, 2007) as well as immigrant adolescents in Norway (Oppedal et al., 2004). Expressive heritage language proficiency among preschool-aged children from low-income Mexican and Chinese immigrant families in the United States has been found to be associated with better socioemotional development (fewer externalizing problems and more prosocial behaviors), with authoritative parenting mediating this relationship (Chung et al., 2019).

In many cases, the assumption in previous research has been that language use and/or proficiency influence family relations, which in turn influence behavioral and mental health. However, it has also been argued that family relations influence language use (or proficiency) rather than the other way around (Tseng & Fuligni, 2000; Wang et al., 2022). As noted above, it is unclear what the directionality of this relationship is (Müller et al., 2020; Oh & Fuligni, 2010).

In a longitudinal framework, family relations may act as a moderator for the relationship between heritage language use and behavioral and mental health. When family relations are strong, increased use of the heritage language can lead to improvements in well-being as it is likely to be related to strengthening ethnic identity (Leyendecker et al., 2018; Oh & Fuligni, 2010) and emotional development (Chen et al., 2012) in a supportive family environment. In families that lack supportive relationships between parents and children, increased use of the heritage language may be more of a reactive development and thus may even result in worse behavioral and mental health outcomes for children. On the side of reduced heritage language use, the negative consequences for behavioral and mental health may be greater for children with close family relations since in these situations the loss of the ethnic heritage may be associated with a greater emotional cost. It can also lead to emotional costs for the parents (De Houwer, 2017). The decreased heritage language use of children in an initially supportive family may also reflect a change in the parent-child relationship (i.e., less family warmth and weaker family cohesion). It has also been suggested that the use of the societal language is less emotional in parent-child interactions (Chen et al., 2012). This could imply that in families with poorer relationships increased use of the societal language may improve the ability to solve emotional issues and thus improve behavioral and mental health of children.

One prior study touches upon this issue of family relations as a moderator: a Norwegian longitudinal study examined heritage language proficiency as part of a measure of ethnic culture competence and did not find family support to moderate the association between ethnic culture competence and mental health (Oppedal et al., 2004).^1^ However, in addition to the fact that the study only measured heritage language proficiency as part of a wider measure of ethnic culture competence, it did not examine externalizing problems and it only had one scale for social support from the family, whereas family relations are measured with three different scales in the current study.

### The Role of the Country Context

Country contexts, which encompass immigration policies related to multiculturalism (e.g., assimilation or integration), can be important for understanding the contingent effect of family relations on the behavioral and mental health consequences of the use of the heritage language within immigrant families. This research considers children of immigrants in the U.K. (or more specifically England), Germany, Sweden and the Netherlands. Research reviewing the association between biculturalism (i.e., the integration strategy within the acculturation framework) and various measures of adaptation has found the link to be weaker in Europe in contrast to the U.S. (Nguyen & Benet-Martínez, 2013). It is also important to note that by far most of the research in this field is based on the U.S. and thus studies from a wider variety of countries are highly important.

Sweden has been known for showing more favorable policy attitudes toward the maintenance of heritage language and multiculturalism compared to the Netherlands with a strong integrationist approach to immigrants and their heritage language use (Vedder & Virta, 2005). In terms of the MIPEX (Migrant Integration Policy Index; see https://www.mipex.eu/ for further information), Sweden has overall the strongest support for integration in the analyzed four countries as well as in the domain of education, which is relevant for the age group studied here. The other three countries do not differ from each other much in terms of the general level of support but the U.K. is slightly behind the Netherlands and Germany in the domain of education. Moreover, these countries differ in their education policies related to teaching heritage languages to immigrant-origin children. Sweden and Germany provide support for maintaining heritage languages among immigrant-origin children through heritage language tuition and in some cases bilingual teaching, whereas the Netherlands and the U.K. do not support the teaching of heritage languages in schools (Eurydice, 2009; European Commission/EACEA/Eurydice, 2017). However, it should also be noted that even in Sweden and Germany support for heritage language maintenance does not extend to all children. Despite institutional support for maintaining heritage languages among children of immigrants, parents play the strongest role in this (Chumak-Horbatsch & Garg, 2006).

Taking these policies together suggests that Sweden is the most favorable context in terms of integration and support for heritage languages, followed by Germany, and the Netherlands and the U.K. are less favorable. Different policies and cultural attitudes related to the language use of immigrants could mean that not only are changes in language use differently associated with changes in behavioral and mental health but also that family relationships play a different role for this association across countries. It has been suggested that a policy climate favoring multiculturalism leads to less conflict between ethnic and mainstream identities and less need to protect the ethnic identity (Yağmur & van de Vijver, 2012). Therefore, increasing (reducing) language use may also be related to better (worse) behavioral and mental health in more (less) favorable contexts. In addition, family relationships may play a stronger role in moderating the relationship between heritage language use and behavioral and mental health in countries where the surrounding society is more hostile toward the use of their heritage language (the Netherlands and the U.K.) than in countries where heritage language use is more supported (Sweden and Germany). Nevertheless, it should be noted that the main aim of the present study was on establishing whether similar processes are at play in the different countries rather than testing whether their strength differs across the countries.

## Current Study

There is a scarcity of research on how changes in acculturation (including but not limited to language use) are related to changes in adaptation outcomes (including but not limited to behavioral and mental health), particularly in a European—or more broadly non-U.S.—context. Moreover, although previous research has touched upon the issue of how social support, including family relations, may moderate this relationship, a more extensive analysis is lacking. Previous research has also predominantly analyzed language use in a scale together with other ethnic practices/orientations or more focus has been placed on language proficiency. While language proficiency and other aspects of ethnic culture and identity are likely to be important too, a direct focus on language is important in its own right, partly because it is difficult to see how language proficiency can develop without continued language use. To address these research gaps, we examine across four European countries (1) how a change in the frequency of speaking the heritage language to family members is associated with internalizing and externalizing problems among immigrant-origin youth, and (2) how family relations moderate this association. We expect that while changes in heritage language use may be rather weakly related to changes in behavioral and mental health, this relationship is likely to be conditioned by the family environment: better family relations offer a context in which increased use of the heritage language within families leads to better behavioral and mental health outcomes. By contrast, increasing the use of the heritage language in a hostile family environment might not necessarily be beneficial for adolescent behavioral and mental health.

## Methods

### Participants and Procedure

This study relies on the first two waves (2010/2011 and 2011/2012) of the cross-national, longitudinal CILS4EU (Children of Immigrants Longitudinal Survey in Four European Countries; Kalter et al., 2016; Kalter et al., 2016) data collected from Germany, the Netherlands, Sweden, and the U.K. (or more specifically England) using a school-based sampling where schools with a higher proportion of children of immigrants were oversampled. In the years of 2010 and 2011 (Wave 1), more than 18,000 majority children and children of immigrants in 480 schools completed a self-completion questionnaire. Participants were followed up in the years of 2011 and 2012 (Wave 2), again with a self-completion questionnaire (Kalter et al., 2016; Kalter et al., 2016).

We restricted our sample to those who completed both waves (4146 in Germany, 3381 in the Netherlands, 4108 in Sweden, 3304 in the U.K.). We excluded children without a migration background (1796 in Germany, 2037 in the Netherlands, 1807 in Sweden, 1464 in the U.K.) and those who lack clear information to identify their immigrant generation (11 in Germany, 8 in the Netherlands, 45 in Sweden, 59 in the U.K.), which yields the valid study sample consisting of 7712 respondents in total (2339 in Germany, 1336 in the Netherlands, 2256 in Sweden, and 1781 the U.K.). We further excluded respondents who have nonresponse to any of the variables included in the models, and sample sizes are different for the models on internalizing problems and the ones on externalizing problems. The majority of missing cases were due to nonresponse to our dependent variables, particularly externalizing problems, in either of waves 1 and 2.^2^ Family relations questions in Germany (family cohesion 13.08%, parental warmth 12.27%, parental monitoring 11.76%) had nonresponse rate higher than 10% largely because family relations questions were not asked in one federal state of Germany (see CILS4EU codebook). Other variables had nonresponse rates lower than 10%. The analysis samples had *N* = 1614 for the internalizing problems, *N* = 1039 for the externalizing problems in Germany; *N* = 1203 for the internalizing problems, *N* = 1129 for the externalizing problems in the Netherlands; *N* = 1794 for the internalizing problems, *N* = 1486 for the externalizing problems in Sweden; *N* = 1359 for the internalizing problems, *N* = 1201 for the externalizing problems in the U.K. The age of the analysis sample ranged between 13 and 17 years old at W1 (M_age_ = 14.8 in Germany, 14.7 in the Netherlands, 14.2 in Sweden, 14.6 in the U.K.) and 50–54% of the sample were female. See Tables 3–6 in Appendix for descriptive statistics for each country.

### Measures

#### Internalizing problems

Following Mood et al. (2016), internalizing problems are measured at waves 1 and 2 by the average response to five questions about how often the respondent feels very worried, depressed (0 “never true”, 1 “rarely true”, 2 “sometimes true”, 3 “often true”), has headaches, stomach aches, and difficulties falling asleep (0 “never/less often”, 1 “once or several times a month”, 2 “once or several times a week”, 3 “every day”). A higher score on the scale indicates that the respondent has more internalizing problems. Cronbach’s alphas for internalizing problems ranged from 0.68 (W1 in the Netherlands) to 0.76 (W2 in Sweden). See Table 7 in the Appendix for more detailed information. The level of internalizing problems at wave 2 is one of our dependent variables and we control for the level at wave 1 in order to focus on change.

#### Externalizing problems

Following Mood et al. (2016), externalizing problems are measured at waves 1 and 2 by the average response to 11 questions about how often the respondent gets angry easily, acts without thinking (0 “never true”, 1 “rarely true”, 2 “sometimes true”, 3 “often true”), argues with a teacher, skips a lesson without permission, comes late to school, drinks alcohol, uses drugs (0 “never/less often”, 1 “once or several times a month”, 2 “once or several times a week”, 3 “every day”), has deliberately damaged things that were not theirs, stolen something from a shop/from someone else, carried a knife or weapon, and been very drunk in the past 3 months (0 “no”, 3 “yes”). A higher score on the scale indicates that the respondent has more externalizing problems. Cronbach’s alphas for externalizing problems ranged from 0.66 (W1 in Germany) to 0.75 (W2 in Germany). See Table 7 in the Appendix for more detailed information. The level of externalizing problems at wave 2 is our other dependent variable and we control for the level at wave 1 in order to focus on change.

#### Use, and change in the use, of the heritage language within the family

The frequency of using the heritage language within the family is measured by the frequency of talking to family in the heritage language at waves 1 and 2 (0 “never”, 1 “sometimes”, 2 “often”, 3 “always”).^3^ If young people did not report speaking another language at home in the filtering question prior to this question, we assigned these respondents to 0 “never”. Our main variable of interest is the change in heritage language frequency in the family between the two waves, which we created by subtracting the heritage language frequency score at wave 1 from the heritage language frequency score at wave 2. A positive score indicates the increased use of the heritage language within the families, while a negative score indicates the decreased use of the heritage language within the families. We also control for the level of heritage language use at wave 1 in our models.

**Fig. 1:**
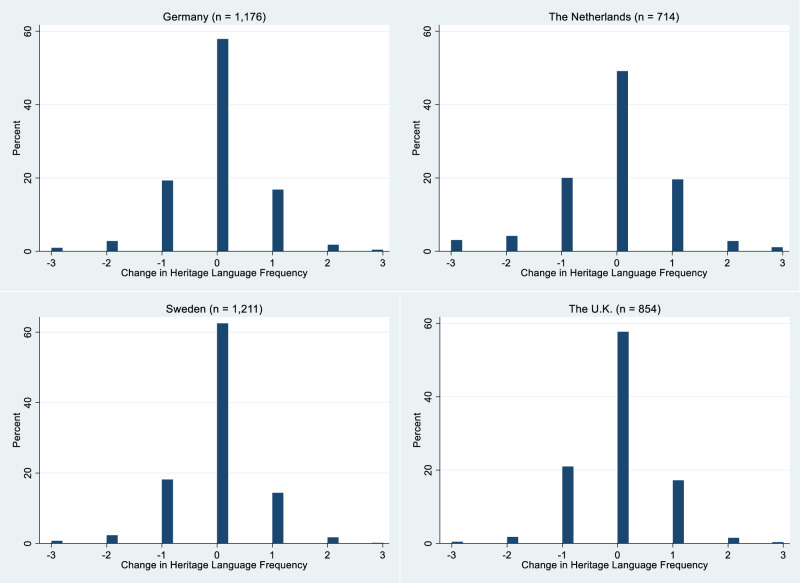
Distribution of change in the frequent use of heritage language in four countries. This graph is based on the sample used in the analysis of the internalizing problems, but includes only those who used heritage language at home in at least one wave

Figure 1 presents the distribution of heritage language frequency change between the two waves, using the samples for the internalizing problems but excluding those who do not report using a heritage language at home in either wave. In general, the majority of respondents did not change their frequency of using the heritage language when talking to family. The distribution around the mean is relatively symmetrical in all four countries, meaning that while there are children who reduced their use of the heritage language at home (a sign of language attrition), despite being a slightly smaller group, there are also substantial numbers who reported increased use of their heritage language.

#### Family cohesion

Following Mood et al. (2017), family cohesion is measured by the average response to the five statements: “We like to spend free time with each other”, “We feel very close to each other”, “It becomes tense when everyone is at home”, “When we are together, the atmosphere is uneasy”, “We fight about small things”. The scale is recoded to range from 0 “never” to 3 “always” (the latter three items are reverse coded) so that a higher score indicates a stronger family cohesion. Cronbach’s alphas ranged from 0.69 (internalizing problems sample in Sweden) to 0.76 (externalizing problems sample in the U.K.). See Table 7 in the Appendix for more detailed information. This variable is centered around the country sample mean. In the figures showing moderating effects, higher family cohesion refers to one standard deviation above the mean and lower family cohesion refers to one standard deviation below the mean.

#### Parental warmth

Parental warmth is measured by the following seven statements (Mood et al., 2017): “Whenever I feel sad, my parents try to comfort me”, “My parents try to help me when I have a problem”, “My parents show me that they love me”, “My parents try to understand what I think and feel”, “My parents often tell me to be quiet”, “My parents are very strict with me even over small things”, “My parents often criticize me”. The scale ranged from 0 “strongly disagree” to 4 “strongly agree”; the latter three items are reverse coded. A higher score indicates higher parental warmth. Cronbach’s alphas ranged from 0.82 (externalizing problems sample in the Netherlands) to 0.84 (externalizing problems sample in Germany). See Table 7 in the Appendix for more detailed information. In the same way as for family cohesion, the variable is centered around the country sample mean and in the figures higher parental warmth refers to one standard deviation above the mean and lower parental warmth to one standard deviation below the mean.

#### Parental monitoring

Parental monitoring is measured by the average response to the following three statements (Mood et al., 2017): “My parents say that I must tell them everything that I do”, “My parents want to know the parents of people I hang out with”, “I always need to tell my parents where I am and what I am doing when I am not at home”. The scale ranges from 0 “strongly disagree” to 4 “strongly agree” so a higher score indicates higher parental monitoring. Cronbach’s alphas ranged from 0.66 (internalizing problems sample in the Netherlands) to 0.72 (internalizing problems sample in Germany). See Table 7 in the Appendix for more detailed information. As with the other two family relations variables, the variable is centered around the country sample mean and in the figures higher parental monitoring refers to one standard deviation above the mean and lower parental monitoring refers to one standard deviation below the mean.

#### Demographics

We also included age, gender (1 = female, 0 = male), immigrant generation, and country of origin which are measured at wave 1 as covariates. Immigrant generation information is included as a categorical variable with three categories: “1st generation (1.25–1.75th)”, “2nd generation (2–2.75th)”, and “3rd generation (3–3.75th)”.^4^ We followed Dollmann et al. (2018) when including the country of origin information. Detailed information on specific origin-country categories can be found in Tables 3–6 in the Appendix. We controlled for school stratum (immigrant proportion) as a categorical variable in our analysis to take into account the oversampling of schools with a higher proportion of students with (non-Western) immigrant backgrounds in the school (Mood et al., 2016, 2017). Tables 3–6 in the Appendix present descriptive statistics of these measures for each country.

### Analysis Plan

**Fig. 2:**
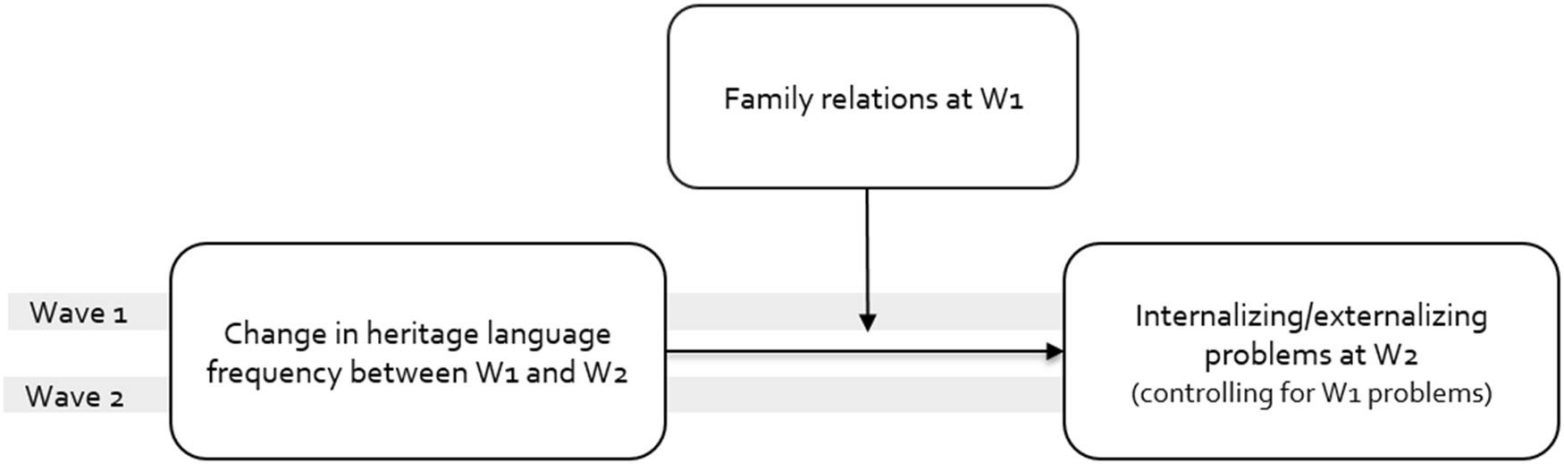
Analytic framework

There is a potential endogeneity issue in understanding the relationship between parenting, heritage language use and internalizing/externalizing problems, and cross-sectional analysis rarely clarifies causality. Taking advantage of the longitudinal nature of the CILS4EU data, we used information from waves 1 and 2, focusing on changes in the frequency of using the heritage language within the family. We examined internalizing and externalizing problems at wave 2 as our dependent variables controlling for internalizing and externalizing problems at wave 1 (i.e., a lagged dependent variable). For our main independent variable, we used changes in frequency that adolescents report speaking the heritage language with their family between the two waves while controlling for the frequency of their heritage language at wave 1. Family relations is measured with three scales (family cohesion, parental warmth, and parental monitoring) at wave 1 and interacted with heritage language frequency change. The scales are all included in all models but the interactions are tested in separate models. This analytic framework is depicted in Fig. 2.

The analysis was conducted with Ordinary Least Squares regressions, separately by country, using Stata 17 version. The results are presented in tables, in addition to which we present the significant interactions in graphs with predicted values, estimated using Stata’s margins-command. For these estimations we use values for family relations one standard deviation above (e.g., higher family cohesion) and one standard deviation below the mean (e.g., lower family cohesion).

## Results

Table 1 present the main regression results of internalizing problems and Table 2 the results for externalizing problems at wave 2 in the four analyzed countries, Germany, the Netherlands, Sweden, and the U.K. (see Tables 8–11 in Appendix for full tables by country).

**Table 1:** OLS regression of internalizing problems at wave 2

Variable	Germany				The Netherlands				Sweden				The U.K.			
	M1a	M2a	M3a	M4a	M1b	M2b	M3b	M4b	M1c	M2c	M3c	M4c	M1d	M2d	M3d	M4d
Change in heritage language frequency	−0.009	−0.009	−0.009	−0.009	−0.021	−0.020	−0.022	−0.023	−0.016	−0.017	−0.015	−0.014	0.044*	0.044*	0.044*	0.034
	(0.018)	(0.018)	(0.018)	(0.018)	(0.019)	(0.019)	(0.019)	(0.019)	(0.021)	(0.021)	(0.021)	(0.021)	(0.023)	(0.023)	(0.022)	(0.023)
Family cohesion	−0.053*	−0.054*	−0.053	−0.054*	−0.013	−0.015	−0.013	−0.012	−0.018	−0.014	−0.020	−0.018	−0.100***	−0.100***	−0.099***	−0.101***
	(0.030)	(0.030)	(0.030)	(0.030)	(0.033)	(0.033)	(0.033)	(0.033)	(0.032)	(0.032)	(0.032)	(0.032)	(0.030)	(0.030)	(0.030)	(0.030)
Parental warmth	−0.010	−0.010	−0.013	−0.011	−0.047	−0.047	−0.047	−0.047	−0.042	−0.043	−0.039	−0.041	0.075**	0.075**	0.072*	0.076**
	(0.022)	(0.022)	(0.022)	(0.022)	(0.026)	(0.026)	(0.026)	(0.026)	(0.026)	(0.026)	(0.027)	(0.026)	(0.025)	(0.025)	(0.025)	(0.025)
Parental monitoring	0.030	0.030	0.029	0.029	0.006	0.006	0.006	0.004	0.005	0.005	0.006	0.002	0.031	0.031	0.031	0.034
	(0.013)	(0.013)	(0.013)	(0.013)	(0.016)	(0.016)	(0.016)	(0.016)	(0.014)	(0.014)	(0.014)	(0.014)	(0.014)	(0.014)	(0.014)	(0.014)
Family cohesion*Change in heritage language frequency		−0.005				−0.043*				0.036				−0.001		
		(0.029)				(0.033)				(0.036)				(0.033)		
Parental warmth*Change in heritage language frequency			−0.022				−0.003				0.015				−0.028	
			(0.021)				(0.024)				(0.028)				(0.030)	
Parental monitoring*Change in Heritage language frequency				−0.025				−0.063**				−0.041*				0.043*
				(0.017)				(0.019)				(0.020)				(0.025)
No. of Obs.	1614	1614	1614	1614	1203	1203	1203	1203	1794	1794	1794	1794	1359	1359	1359	1359
Adjusted R-Squared	0.397	0.397	0.398	0.398	0.431	0.432	0.430	0.434	0.385	0.386	0.385	0.387	0.449	0.448	0.449	0.450

Standardized coefficients appear above standard errors in parenthesis. All models are controlled for internalizing problems at wave 1, heritage language frequency at wave 1, age at wave 1, gender, immigrant generation, country of origin, and stratum. Family relations variables (family cohesion, parental warmth, parental monitoring) are centered around the country sample mean. See Tables 8–11 in Appendix for the table with full information by country

*p < 0.05; **p < 0.01; ***p < 0.001 (two-tailed tests)

**Table 2:** OLS regression of externalizing problems at wave 2

Variable	Germany				The Netherlands				Sweden				The U.K.			
	M5a	M6a	M7a	M8a	M5b	M6b	M7b	M8b	M5c	M6c	M7c	M8c	M5d	M6d	M7d	M8d
Change in heritage language frequency	−0.063*	−0.061*	−0.062*	−0.060*	−0.013	−0.013	−0.013	−0.014	−0.008	−0.007	−0.008	−0.006	−0.015	−0.005	−0.011	−0.005
	(0.017)	(0.017)	(0.017)	(0.017)	(0.014)	(0.014)	(0.014)	(0.014)	(0.014)	(0.014)	(0.014)	(0.014)	(0.016)	(0.016)	(0.016)	(0.017)
Family cohesion	−0.014	−0.017	−0.012	−0.014	0.026	0.026	0.026	0.026	−0.035	−0.040	−0.035	−0.036	−0.064*	−0.072*	−0.060	−0.064*
	(0.027)	(0.027)	(0.027)	(0.027)	(0.025)	(0.025)	(0.025)	(0.025)	(0.021)	(0.021)	(0.021)	(0.021)	(0.022)	(0.021)	(0.021)	(0.022)
Parental warmth	0.016	0.014	0.007	0.015	−0.054	−0.054	−0.054	−0.055	−0.029	−0.029	−0.029	−0.029	0.041	0.043	0.031	0.040
	(0.020)	(0.020)	(0.020)	(0.020)	(0.020)	(0.020)	(0.020)	(0.020)	(0.017)	(0.017)	(0.018)	(0.017)	(0.018)	(0.018)	(0.018)	(0.018)
Parental monitoring	0.022	0.020	0.019	0.019	−0.023	−0.023	−0.023	−0.023	−0.043*	−0.043*	−0.043*	−0.047*	−0.043	−0.043	−0.045*	−0.045*
	(0.012)	(0.012)	(0.012)	(0.012)	(0.012)	(0.012)	(0.012)	(0.012)	(0.009)	(0.009)	(0.009)	(0.009)	(0.011)	(0.011)	(0.011)	(0.011)
Family cohesion*Change in heritage language frequency		−0.059*				0.008				−0.036				−0.080***		
		(0.025)				(0.026)				(0.022)				(0.023)		
Parental warmth*Change in heritage language frequency			−0.076**				0.003				0.000				−0.077***	
			(0.019)				(0.018)				(0.017)				(0.021)	
Parental monitoring*Change in heritage language frequency				−0.040				−0.034				−0.030				−0.034
				(0.016)				(0.014)				(0.013)				(0.017)
No. of Obs.	1039	1039	1039	1039	1129	1129	1129	1129	1486	1486	1486	1486	1201	1201	1201	1201
Adjusted R-Squared	0.376	0.379	0.381	0.377	0.367	0.366	0.366	0.367	0.415	0.416	0.415	0.416	0.421	0.427	0.426	0.421

Standardized coefficients appear above standard errors in parenthesis. All models are controlled for externalizing problems at wave 1, heritage language frequency at wave 1, age at wave 1, gender, immigrant generation, country of origin, and stratum. Family relations variables (family cohesion, parental warmth, parental monitoring) are centered around the country sample mean. See Tables 8–11 in Appendix for the table with full information by country

*p < 0.05; **p < 0.01; ***p < 0.001 (two-tailed tests)

### The Use of Heritage Language, Family Relations, and Behavioral and Mental Health

In terms of the main effect of change in heritage language for internalizing problems (Models 1a, 1b, 1c and 1d), Table 1 shows that there was a statistically significant, positive main effect only in the U.K. (Model 1d); increased use of heritage language within the family between the waves was associated with more internalizing problems at wave 2. Four statistically significant interaction effects are also evident in Table 1: for family cohesion in the Netherlands (Model 2b), for parental monitoring in the Netherlands (Model 4b) and Sweden (Model 4c) as well as in the U.K. (Model 4d). It should be noted that the interaction for the U.K. is in the opposite direction to those in the Netherlands and Sweden.

**Fig. 3:**
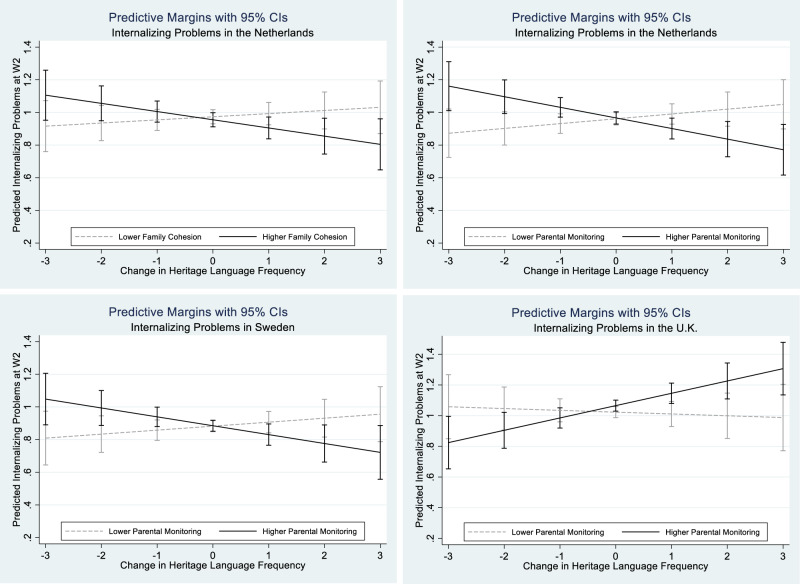
The moderating effect of family relations on change in heritage language frequency and internalizing problems at wave 2. The upper left graph is calculated based on M2b of Table 1, and the upper right graph based on M4b of Table 1. The lower left graph is calculated based on M4c of Table 1, and the lower right graph based on M4d of Table 1. Black solid lines present higher family cohesion or parental monitoring (one standard deviation above the mean score of family relations), and gray dashed lines present lower family cohesion or parental monitoring (one standard deviation below the mean score of family relations)

Figure 3 visualizes these four interactions. The upper left panel displays the negative association between the change in heritage language frequency and wave 2 internalizing problems for adolescents with a higher level of family cohesion (one standard deviation above the mean; beta = −0.050, *p* = 0.047) and a slightly positive, but not statistically significant association for adolescents with a lower level of family cohesion (one standard deviation below the mean; beta = 0.019, *p* = 0.460) in the Netherlands. Likewise, the upper right panel displays these same associations for parental monitoring in the Netherlands (beta = −0.065, *p* = 0.010 for higher parental monitoring; beta = 0.029, *p* = 0.230 for lower parental monitoring) and the lower left panel for parental monitoring in Sweden (beta = −0.054, *p* = 0.043 for higher parental monitoring; beta = 0.024, *p* = 0.378 for lower parental monitoring). Finally, the lower right panel displays the opposite pattern for parental monitoring in the U.K.: increases in heritage language use were associated with more internalizing problems for children with higher parental monitoring (beta = 0.081, *p* = 0.004), but the association was not significant for children with lower parental monitoring (beta = −0.012, *p* = 0.744).

Turning to Table 2 and the main effect of change in heritage language use for externalizing problems (Models 5a, 5b, 5c, 5d), this was only significant in one country: Germany. Increased use of the heritage language was associated with reduced externalizing problems at wave 2. Again, four statistically significant interactions are evident in Table 2: for family cohesion and parental warmth in Germany (Models 6a and 7a), and for the same two measures in the U.K. (Models 6d and 7d).

**Fig. 4:**
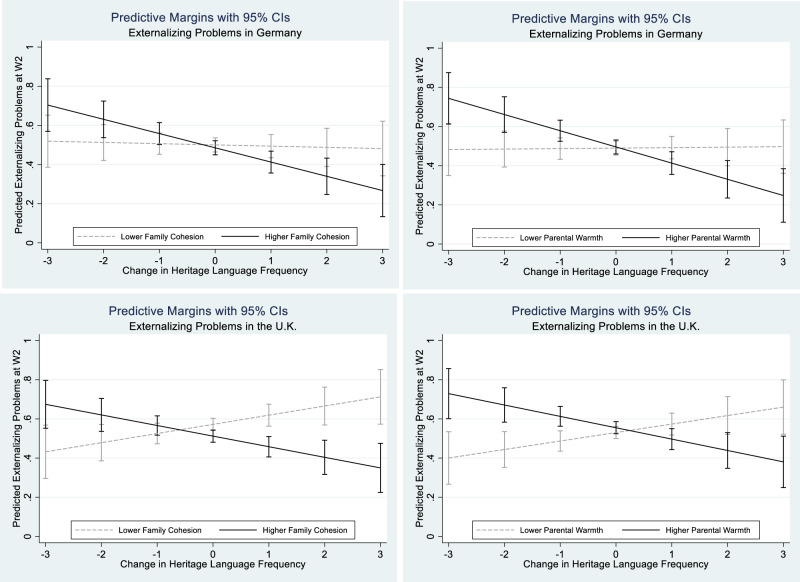
The moderating effect of family relations on change in heritage language frequency and externalizing problems at wave 2. The upper left graph is calculated based on M6a of Table 2, and the upper right graph based on M7a of Table 2. The lower left graph is calculated based on M6d of Table 2, and the lower right graph based on M7d of Table 2. Black solid lines present higher family cohesion or parental warmth (one standard deviation above the mean score of family relations), and gray dashed lines present lower family cohesion or parental warmth (one standard deviation below the mean score of family relations)

These four interactions are visualized in Fig. 4. The upper left panel is the interaction between family cohesion and changes in heritage language use in Germany whereas in the upper right panel is the interaction for parental warmth in the same country. In both cases, heritage language use was not associated with externalizing problems for children reporting low levels of family cohesion (beta = −0.006, *p* = 0.779) or parental warmth (beta = 0.002, *p* = 0.911), but increased use was associated with fewer externalizing problems for those with high family cohesion (beta = −0.073, *p* = 0.001) or parental warmth (beta = −0.083, *p* = 0.000)—and reduced use with more problems. The lower panels show these same relationships for the U.K. In this country context, the patterns for high family cohesion (lower left panel; beta = −0.054, *p* = 0.008) or parental warmth (lower right panel; beta = −0.058, *p* = 0.007) also hold, but in addition increased heritage language use was associated with more externalizing problems for those with low family cohesion (beta = 0.047, *p* = 0.040) or parental warmth (beta = 0.044, *p* = 0.054), though this becomes statistically significant at slightly lower level of parental warmth than one standard deviation below the mean.

Overall, increased use of the heritage language within families was found to be related to fewer externalizing problems for children with better family relations (in terms of family cohesion and parental warmth) in Germany and the U.K. It is also the case that reduced use of the heritage language in these kinds of families tended to be associated with more externalizing problems. For children with lower family cohesion and parental warmth, increasing the use of their heritage language when talking with family members did not help behavioral and mental health in Germany and was associated with higher levels of externalizing problems in the U.K., and at the same time reduced use with lower levels of externalizing problems. We did not find similar results for the Netherlands or Sweden (although the interaction coefficient for family cohesion in Sweden was in the same direction).

As for internalizing problems, in the Netherlands and Sweden, increased use of the heritage language within families was related to fewer internalizing problems for children with higher parental monitoring (and in the Netherlands also higher family cohesion), but at the same time reduced use to more internalizing problems in these types of families. On the other hand, the opposite result was found in the U.K.: increased use of the heritage language within families was related to more internalizing problems for children with higher parental monitoring. The German results for parental monitoring were in the same direction as for the Netherlands and Sweden but not statistically significant.

### Sensitivity Analysis

As a sensitivity analysis, we conducted the regressions without controlling for heritage language frequency at wave 1 in the models and the interaction results remained the same. To test an alternative modeling approach, we also ran the Ordinary Least Squares regressions using changes in internalizing and externalizing problems between two waves as our dependent variables. As we did for the change in their heritage language frequency, we created the change in our dependent variables by subtracting the wave 1 scores from wave 2 scores. The interaction results remained the same with only two exceptions: the statistically significant interaction effect of family cohesion on the change in the internalizing problems in the Netherlands disappeared in the sensitivity analysis while the interaction effect of family cohesion on the change in externalizing problems in Sweden that was insignificant in our main analysis became statistically significant at *p* < 0.05. Other than these exceptions, we found the findings remained robust: the increased use of the heritage language within families is related to fewer internalizing problems for children with higher parental monitoring (the Netherlands and Sweden) and fewer externalizing problems for children with higher family cohesion and parental warmth (Germany and the U.K.). For children with lower family cohesion and parental warmth, increasing the use of their heritage language when talking with family members was not helpful for behavioral problems.

## Discussion

There are a number of gaps in research related to the association between the acculturation of children of immigrants, including their language use at home, and their adaptation, including behavioral and mental health. One is that much of this research is cross-sectional, the second that it tends to be based on samples from the U.S., and the third that it has not focused on how family relations moderate this relationship. Yet a more multifaceted understanding of how changes in acculturation are associated with changes in the adaptation of immigrant-origin adolescents is vital as this group makes up a substantial proportion of young people in many countries around the world and the challenges that they face are in some respects distinct from their majority peers. This study examined whether the relationship between heritage language use within immigrant families and children’s behavioral and mental health is contingent on family relations as a proximal environment for child development. The current study tested this in a longitudinal framework, assessing how the change in heritage language use is related to change in behavioral and mental health in Germany, the Netherlands, Sweden, and the U.K. Whereas previous research has emphasized the development of the societal language as well as heritage language proficiency, this study contributes to the literature through its focus on heritage language use as a precondition for heritage language proficiency to develop and as a more direct measure of ethnic practices within families.

The results of the current study suggest that overall changes in heritage language use are not directly related to changes in behavioral and mental health. However, family relations positively moderated the relationship between change in their heritage language use within families and adolescents’ behavioral and mental health in all four countries though in somewhat different ways. Increased use of the heritage language when communicating with family was associated with reduced internalizing problems for children with higher parental monitoring in the Netherlands and Sweden. In contrast, children from families with higher parental monitoring who increase the use of their heritage language over time displayed reductions in their mental well-being in the U.K. On the other hand, increased use of the heritage language was associated with reduced externalizing problems for children with better family relations (higher family cohesion and parental warmth) in Germany and the U.K. In contrast, children from families with lower family cohesion and parental warmth who increased their heritage language use over time even displayed increase behavioral problems in the U.K.

### Changes in Behavioral and Mental Health and their Association with Changes in Heritage Language Use and Family Relations

Previous research has acknowledged how intertwined language use within families, family relationships and children’s behavioral and mental health are (e.g., Butcher, 2008; Müller et al., 2020). Most previous research has approached this from the point of view of heritage language proficiency and family relations or parenting styles as a mediator of the relationship between language and children’s well-being (Birman & Taylor-Ritzler, 2007; Chung et al., 2019; Oppedal et al., 2004). Acculturation gaps between parents and children are associated with poorer family relations and both directly and indirectly to poorer behavioral and mental health (e.g., Chen et al., 2014; Costigan & Dokis, 2006; Schwartz et al., 2016). In some families children may be expected to be translators or language brokers for their parents, which can create tension and conflict between parents and children (Foner & Dreby, 2011; Shen et al., 2022), and result in greater behavioral and mental health problems for the children (Belhadj Kouider et al., 2015; Shen et al., 2022).

In our results, parental warmth and family cohesion seemed to work in a similar way in moderating the relationship between language use and externalizing problems. These two aspects of family intimacy are thus important preconditions for increased language use to be associated with fewer behavioral problems. It has been suggested that heritage language use is more frequent and more efficient for expressing emotions between parents and children (Chen et al., 2012) and thus more emotional talk is associated with fewer problem behaviors only when it takes place in a supportive environment. In addition, since the use of the societal language may be better for solving emotional problems (Chen et al., 2012), this may also explain the U.K. findings that reduced use of the heritage language was associated with fewer behavioral problems in less warm and cohesive families, where the need to solve emotional problems may be greater.

On the other side, a supportive family environment also means that reduced language use is associated with greater behavioral problems. In these cases a distancing from ethnic cultural practices seemed to lead to more problem behaviors, particularly when contrasted with less warm and cohesive families. Children turning away from the use of the heritage language may cause distress to parents (De Houwer, 2017) which may also then increase children’s problem behavior. One part of this may also be that the reduced use of the heritage language in these former families is also linked to a deterioration of family relations, which can also lead to more externalizing problems (see Oppedal et al., 2004, for evidence on change in family support mediating the relationship between change in ethnic cultural competence and change in mental health). However, we were not able to test this type of mediation since we do not have measures of the family environment at wave 2. Moreover, other research has tended to find rather inconclusive evidence of reduced heritage language proficiency among children leading to poorer family relationships (Park et al., 2012; Tseng & Fuligni, 2000).

In contrast, in the relationship between language use and internalizing problems, the moderating family relations variable was mostly parental monitoring. Setting aside the U.K. results, which may be a false positive and should be studied further, families with higher parental monitoring seemed to provide the best conditions for increased language use to lead to fewer internalizing problems. This type of parenting thus provides a better environment for stronger ethnic cultural practices to lead to better mental health than more lenient parenting. Again though, it should be acknowledged that reduced language use was associated with more mental health issues when parental monitoring was high. In these cases, reduced heritage language use with parents may be a reaction to what the youth perceive to be too strict parenting but which may then lead to greater internalizing problems. This also reflects previous discussions that parental monitoring is likely to mainly be beneficial when children do not perceive it as being coercive, i.e., when it is in line with authoritative parenting rather than authoritarian parenting (Mood et al., 2017).

Despite the challenges that language issues can cause, immigrant families in many cases hold on to values that maintain strong family relationships, partly as a means to support family well-being in the context of acculturation stressors (Foner & Dreby, 2011; Liebkind & Jasinskaja-Lahti, 2000). The findings of this study suggests that building a positive and healthy family environment can be an important condition for the increased use of the heritage language within immigrant families to lead to behavioral health benefits. It is also suggested that family environments with high levels of parental monitoring are necessary for the increased use of the heritage language to lead to mental health benefits.

### Differences Across Countries

With regard to the country patterns, the moderating effects that we found were not in line with the initial expectation that family relations would be more important in countries with less support for immigrant integration and particularly heritage language maintenance (i.e., the Netherlands and the U.K. versus Germany and Sweden). The results rather suggest that similar processes are at play in the Netherlands and Sweden on the one hand and Germany and the U.K. on the other. At the same time it should be noted that some indications of similar processes were found across these country divisions though they were not statistically significant. Overall, these country differences thus warrant further investigation. One possibility for this would be to look into more detail whether there are differences across ethnic minority groups in these countries and thus whether compositional differences in this regard may explain some of the country differences. Above all, the opposing pattern for the U.K. in terms of the moderating effect of parental monitoring as well as the overall negative association between language use and mental health should be analyzed with different data.

### Limitations and Future Research Directions

The present study has limitations that suggest research directions for future studies. First, while the findings suggest the moderating role of family relations in the relationship between the use of their heritage language and adolescent behavioral and mental health, the present study was not able to examine the possible mechanisms underlying this relationship. Therefore, future research should examine possible explanations, including the links to parents’ societal language proficiency, to better understand the conditions in which the behavioral and mental health benefits of their heritage language use can be materialized. As mentioned above, possible ethnic minority group differences should also be examined.

The CILS4EU data used in this study do not have measures for family relations at wave 2, which limits our analysis of the longitudinal effect of family relations on changes in adolescent behavioral and mental health problems.^5^ Some of these relationships may be due to ceiling and floor effects: for those with very high levels of family relations, the only possibilities are for stability or deterioration—and the latter is then likely to be associated with increased behavioral and mental health problems—and vice versa for those with low levels of family relations. Future studies with longitudinal data should clarify these possibilities.

## Conclusion

Prior scholarship has largely focused on the role of societal language or heritage language proficiency in adolescent adaptation outcomes, particularly in the U.S. context and/or using cross-sectional data. To address these research gaps, the current study expands the scope of the scholarship to the understudied role of heritage language use for behavioral and mental health, and examines how family relations provide different conditions for these relationships to play out longitudinally in four European countries. The findings of this study show that increased use of heritage language is associated with fewer externalizing problems only in families with greater family cohesion and parental warmth (in Germany and the U.K.) and with fewer internalizing problems only in families with higher parental monitoring (in the Netherlands and Sweden). Adolescents with healthy and positive family relationships can benefit from the increased use of their heritage language in terms of reduced internalizing and externalizing problems, but at the same time reduced heritage language use is associated with more internalizing and externalizing problems in these types of families. These findings provide more nuance to work on processes of acculturation and how they are intertwined with family relationships, and further offer important implications for family and education policies targeting the well-being of adolescents with an immigrant background. The findings suggest that in order to support the behavioral and mental health of children of immigrants, it is important to pay attention to both their opportunities for heritage language maintenance as well as support immigrant families to build healthy and positive relationships.

## Appendix. Descriptive Statistics and the OLS Regression Results with Full Information by Country

Tables 3–11

**Table 3:** Descriptive statistics for Germany

Variable	Internalizing problems (*N* = 1614)				Externalizing problems (*N* = 1039)			
	Mean	Std. Dev.	Min	Max	Mean	Std. Dev.	Min	Max
Internalizing/externalizing problems at W1	1.06	0.57	0	3	0.46	0.36	0	1.91
Internalizing/externalizing problems at W2	1.06	0.60	0	3	0.49	0.43	0	3
Change in heritage language frequency	−0.04	0.70	−3	3	−0.02	0.67	−3	3
Frequency of heritage language use at W1	1.53	1.22	0	3	1.55	1.21	0	3
Frequency of heritage language use at W2	1.49	1.21	0	3	1.53	1.20	0	3
Age at W1	14.78	0.75	13	17	14.79	0.74	13	17
Female	0.50	0.50	0	1	0.50	0.50	0	1
*Immigrant generation*								
1st generation	0.17	0.38	0	1	0.17	0.38	0	1
2nd generation	0.62	0.49	0	1	0.64	0.48	0	1
3rd generation	0.21	0.41	0	1	0.19	0.40	0	1
*Country of origin*								
Southern and Other Europe (incl. Greece)	0.10	0.30	0	1	0.09	0.29	0	1
Turkey	0.27	0.44	0	1	0.28	0.45	0	1
Former Soviet Union	0.13	0.33	0	1	0.13	0.33	0	1
Poland	0.11	0.31	0	1	0.11	0.32	0	1
Former Yugoslavia	0.07	0.26	0	1	0.06	0.25	0	1
Asia	0.08	0.27	0	1	0.09	0.28	0	1
Italy	0.06	0.24	0	1	0.06	0.23	0	1
Eastern Europe	0.06	0.23	0	1	0.05	0.21	0	1
Northern and Other Africa (incl. Lebanon)	0.06	0.23	0	1	0.05	0.22	0	1
Northern and Latin America	0.03	0.17	0	1	0.03	0.17	0	1
Unknown country of origin	0.04	0.21	0	1	0.05	0.21	0	1
Family cohesion (centered)	0.00	0.55	−2.03	0.97	0.00	0.55	−2.03	0.97
Parental warmth (centered)	0.00	0.72	−2.83	1.17	0.00	0.74	−2.81	1.19
Parental monitoring (centered)	0.00	0.93	−2.17	1.83	0.00	0.94	−2.16	1.84
*Stratum (immigrant proportion)*								
0–10%	0.11	0.31	0	1	0.12	0.33	0	1
10–30%	0.31	0.46	0	1	0.24	0.42	0	1
30–60%	0.28	0.45	0	1	0.33	0.47	0	1
60–100%	0.31	0.46	0	1	0.31	0.46	0	1

**Table 4:** Descriptive statistics for the Netherlands

Variable	Internalizing problems (*N* = 1203)				Externalizing problems (*N* = 1129)			
	Mean	Std. Dev.	Min	Max	Mean	Std. Dev.	Min	Max
Internalizing/externalizing problems at W1	0.93	0.57	0	3	0.44	0.38	0	2.73
Internalizing/externalizing problems at W2	0.97	0.61	0	3	0.47	0.42	0	3
Change in heritage language frequency	−0.05	0.79	−3	3	−0.05	0.78	−3	3
Frequency of heritage language use at W1	1.10	1.16	0	3	1.11	1.16	0	3
Frequency of heritage language use at W2	1.05	1.16	0	3	1.06	1.16	0	3
Age at W1	14.67	0.69	13	17	14.67	0.69	13	17
Female	0.54	0.50	0	1	0.54	0.50	0	1
*Immigrant generation*								
1st generation	0.15	0.36	0	1	0.16	0.36	0	1
2nd generation	0.57	0.50	0	1	0.57	0.50	0	1
3rd generation	0.28	0.45	0	1	0.28	0.45	0	1
*Country of origin*								
Southern, Western, and Other Europe (incl. Germany)	0.17	0.38	0	1	0.16	0.37	0	1
Turkey	0.13	0.33	0	1	0.13	0.34	0	1
Morocco	0.14	0.35	0	1	0.14	0.35	0	1
Suriname	0.13	0.34	0	1	0.13	0.33	0	1
Indonesia	0.10	0.29	0	1	0.10	0.30	0	1
Netherlands Antilles	0.07	0.25	0	1	0.07	0.26	0	1
Asia	0.11	0.31	0	1	0.11	0.32	0	1
Africa	0.06	0.24	0	1	0.06	0.24	0	1
Northern and Latin America	0.04	0.19	0	1	0.04	0.20	0	1
Unknown country of origin	0.06	0.23	0	1	0.05	0.22	0	1
Family cohesion (centered)	0.00	0.54	−2.19	0.81	0.00	0.52	−2.21	0.79
Parental warmth (centered)	0.00	0.64	−2.49	1.08	0.00	0.64	−2.50	1.07
Parental monitoring (centered)	0.00	0.88	−2.00	2.00	0.00	0.88	−1.99	2.01
*Stratum (immigrant proportion)*								
0–10%	0.11	0.31	0	1	0.11	0.31	0	1
10–30%	0.28	0.45	0	1	0.28	0.45	0	1
30–60%	0.36	0.48	0	1	0.36	0.48	0	1
60–100%	0.26	0.44	0	1	0.26	0.44	0	1

**Table 5:** Descriptive statistics for Sweden

Variable	Internalizing problems (*N* = 1794)				Externalizing problems (*N* = 1486)			
	Mean	Std. Dev.	Min	Max	Mean	Std. Dev.	Min	Max
Internalizing/externalizing problems at W1	0.77	0.59	0	3	0.41	0.36	0	2.36
Internalizing/externalizing problems at W2	0.89	0.65	0	3	0.45	0.40	0	3
Change in heritage language frequency	−0.05	0.62	−3	3	−0.05	0.62	−3	3
Frequency of heritage language use at W1	1.41	1.24	0	3	1.34	1.24	0	3
Frequency of heritage language use at W2	1.36	1.24	0	3	1.29	1.23	0	3
Age at W1	14.17	0.43	13	17	14.17	0.43	13	17
Female	0.53	0.50	0	1	0.54	0.50	0	1
*Immigrant generation*								
1st generation	0.20	0.40	0	1	0.18	0.39	0	1
2nd generation	0.57	0.49	0	1	0.57	0.50	0	1
3rd generation	0.23	0.42	0	1	0.25	0.43	0	1
*Country of origin*								
Southern Europe, Germany, Denmark, and Norway	0.11	0.31	0	1	0.12	0.32	0	1
Former Yugoslavia	0.13	0.33	0	1	0.12	0.33	0	1
Finland	0.11	0.32	0	1	0.13	0.33	0	1
Iraq	0.07	0.25	0	1	0.06	0.23	0	1
Turkey	0.07	0.25	0	1	0.07	0.25	0	1
Somalia	0.03	0.16	0	1	0.02	0.16	0	1
Middle East and Northern Africa (incl. Lebanon, Syria, Iran)	0.11	0.32	0	1	0.11	0.32	0	1
Eastern and Other Africa	0.04	0.19	0	1	0.03	0.18	0	1
Asia	0.12	0.32	0	1	0.11	0.31	0	1
Eastern and Other Europe (incl. Poland)	0.09	0.29	0	1	0.09	0.29	0	1
Latin and Northern America	0.05	0.21	0	1	0.05	0.22	0	1
Unknown country of origin	0.08	0.28	0	1	0.09	0.28	0	1
Family cohesion (centered)	0.00	0.53	−2.29	0.71	0.00	0.54	−2.29	0.71
Parental warmth (centered)	0.00	0.63	−3.19	0.81	0.00	0.64	−3.18	0.82
Parental monitoring (centered)	0.00	0.89	−1.89	2.11	0.00	0.91	−1.89	2.11
*Stratum (immigrant proportion)*								
0–10%	0.09	0.28	0	1	0.09	0.29	0	1
10–30%	0.25	0.43	0	1	0.27	0.44	0	1
30–60%	0.30	0.46	0	1	0.30	0.46	0	1
60–100%	0.36	0.48	0	1	0.34	0.47	0	1

**Table 6:** Descriptive statistics for the U.K.

Variable	Internalizing problems (*N* = 1359)				Externalizing problems (*N* = 1201)			
	Mean	Std. Dev.	Min	Max	Mean	Std. Dev.	Min	Max
Internalizing/externalizing problems at W1	1.01	0.59	0	3	0.59	0.43	0	2.91
Internalizing/externalizing problems at W2	1.04	0.63	0	3	0.54	0.42	0	3
Change in heritage language frequency	−0.03	0.61	−3	3	−0.02	0.61	−3	3
Frequency of heritage language use at W1	1.07	1.14	0	3	1.06	1.15	0	3
Frequency of heritage language use at W2	1.04	1.13	0	3	1.03	1.13	0	3
Age at W1	14.64	0.52	13	17	14.65	0.52	13	17
Female	0.50	0.50	0	1	0.52	0.50	0	1
*Immigrant generation*								
1st generation	0.24	0.43	0	1	0.23	0.42	0	1
2nd generation	0.50	0.50	0	1	0.49	0.50	0	1
3rd generation	0.26	0.44	0	1	0.27	0.44	0	1
*Country of origin*								
Southern and Other Europe (incl. Ireland)	0.14	0.35	0	1	0.15	0.36	0	1
Pakistan	0.16	0.36	0	1	0.15	0.35	0	1
Asia (incl. Bangladesh)	0.12	0.33	0	1	0.12	0.33	0	1
India	0.14	0.34	0	1	0.14	0.35	0	1
Eastern and Western Africa (incl. Nigeria)	0.11	0.31	0	1	0.11	0.31	0	1
Latin America and Caribbean (incl. Jamaica)	0.09	0.28	0	1	0.09	0.28	0	1
Other Africa	0.04	0.20	0	1	0.04	0.19	0	1
Eastern Europe	0.02	0.15	0	1	0.02	0.14	0	1
Other (Northern America and Oceania)	0.02	0.15	0	1	0.02	0.15	0	1
Unknown country of origin	0.16	0.37	0	1	0.16	0.37	0	1
Family cohesion (centered)	0.00	0.60	−2.13	0.87	0.00	0.60	−2.13	0.87
Parental warmth (centered)	0.00	0.70	−2.80	1.20	0.00	0.70	−2.80	1.20
Parental monitoring (centered)	0.00	0.91	−2.24	1.76	0.00	0.90	−2.24	1.76
*Stratum (immigrant proportion)*								
0–10%	0.05	0.22	0	1	0.05	0.22	0	1
10–30%	0.25	0.43	0	1	0.25	0.43	0	1
30–60%	0.24	0.43	0	1	0.24	0.43	0	1
60–100%	0.37	0.48	0	1	0.36	0.48	0	1
Independent schools	0.10	0.29	0	1	0.10	0.30	0	1

**Table 7:** Cronbach’s alphas for composite measures

	Germany		The Netherlands		Sweden		The U.K.	
Variable	Internal	External	Internal	External	Internal	External	Internal	External
Internalizing problems/Externalizing problems at W1	0.69	0.66	0.68	0.73	0.73	0.72	0.69	0.74
Internalizing problems/Externalizing problems at W2	0.72	0.75	0.72	0.75	0.76	0.74	0.73	0.73
Family cohesion	0.71	0.71	0.72	0.71	0.69	0.71	0.76	0.76
Parental warmth	0.83	0.84	0.82	0.82	0.82	0.82	0.82	0.82
Parental monitoring	0.72	0.71	0.66	0.67	0.68	0.69	0.70	0.69

**Table 8:** OLS regression of internalizing and externalizing problems at wave 2 in Germany

	Internalizing problems				Externalizing problems			
Variable	M1a	M2a	M3a	M4a	M5a	M6a	M7a	M8a
Internalizing/Externalizing problems at W1	0.530***	0.530***	0.530***	0.529***	0.591***	0.590***	0.590***	0.591***
	(0.023)	(0.023)	(0.023)	(0.023)	(0.032)	(0.032)	(0.032)	(0.032)
Change in heritage language use	−0.009	−0.009	−0.009	−0.009	−0.063*	−0.061*	−0.062*	−0.060*
	(0.018)	(0.018)	(0.018)	(0.018)	(0.017)	(0.017)	(0.017)	(0.017)
Heritage language use at W1	−0.046	−0.045	−0.045	−0.046	−0.122***	−0.119**	−0.117**	−0.122***
	(0.014)	(0.014)	(0.014)	(0.014)	(0.013)	(0.013)	(0.013)	(0.013)
Age	0.040	0.040	0.040	0.039	0.013	0.014	0.013	0.012
	(0.017)	(0.017)	(0.017)	(0.017)	(0.015)	(0.015)	(0.015)	(0.015)
Female	0.160***	0.160***	0.161***	0.160***	−0.067**	−0.065*	−0.065*	−0.067**
	(0.026)	(0.026)	(0.026)	(0.026)	(0.022)	(0.022)	(0.022)	(0.022)
*Immigrant generation* (ref = 1st generation)								
2nd generation	0.029	0.030	0.031	0.029	−0.088*	−0.081*	−0.078*	−0.088*
	(0.036)	(0.037)	(0.037)	(0.036)	(0.033)	(0.033)	(0.033)	(0.033)
3rd generation	−0.032	−0.031	−0.031	−0.033	−0.101*	−0.097*	−0.095*	−0.101*
	(0.054)	(0.054)	(0.054)	(0.054)	(0.050)	(0.050)	(0.050)	(0.050)
*Country of origin*^a^								
Turkey	−0.004	−0.005	−0.007	−0.005	−0.042	−0.049	−0.052	−0.045
	(0.047)	(0.048)	(0.048)	(0.047)	(0.043)	(0.043)	(0.043)	(0.043)
Former Soviet Union	−0.002	−0.002	−0.003	−0.002	−0.031	−0.035	−0.037	−0.031
	(0.053)	(0.053)	(0.053)	(0.053)	(0.049)	(0.049)	(0.049)	(0.049)
Poland	−0.033	−0.033	−0.034	−0.032	−0.015	−0.019	−0.020	−0.015
	(0.052)	(0.052)	(0.052)	(0.052)	(0.048)	(0.048)	(0.048)	(0.048)
Former Yugoslavia	−0.007	−0.007	−0.009	−0.007	−0.005	−0.010	−0.013	−0.006
	(0.059)	(0.059)	(0.059)	(0.059)	(0.056)	(0.056)	(0.056)	(0.056)
Asia	0.003	0.003	0.002	0.003	−0.053	−0.056	−0.054	−0.055
	(0.058)	(0.058)	(0.058)	(0.058)	(0.052)	(0.052)	(0.052)	(0.052)
Italy	−0.017	−0.017	−0.018	−0.017	−0.048	−0.049	−0.051	−0.049
	(0.061)	(0.061)	(0.061)	(0.061)	(0.057)	(0.057)	(0.056)	(0.057)
Eastern Europe	0.001	0.001	0.001	0.001	−0.047	−0.047	−0.047	−0.047
	(0.064)	(0.064)	(0.064)	(0.064)	(0.061)	(0.061)	(0.061)	(0.061)
Northern and Other Africa (incl. Lebanon)	−0.036	−0.036	−0.037	−0.035	−0.038	−0.040	−0.043	−0.035
	(0.064)	(0.064)	(0.064)	(0.064)	(0.060)	(0.060)	(0.059)	(0.060)
Northern and Latin America	0.004	0.005	0.004	0.004	−0.027	−0.028	−0.029	−0.030
	(0.077)	(0.077)	(0.077)	(0.077)	(0.071)	(0.071)	(0.071)	(0.071)
Unknown country of origin	0.013	0.013	0.012	0.013	−0.005	−0.006	−0.007	−0.006
	(0.071)	(0.071)	(0.071)	(0.071)	(0.065)	(0.065)	(0.065)	(0.065)
*Stratum* (ref = 0–10%)								
10–30%	−0.068*	−0.069*	−0.069*	−0.067*	0.000	−0.004	−0.006	0.004
	(0.042)	(0.042)	(0.042)	(0.042)	(0.038)	(0.038)	(0.038)	(0.038)
30–60%	−0.030	−0.030	−0.031	−0.030	−0.007	−0.010	−0.012	−0.005
	(0.044)	(0.044)	(0.044)	(0.044)	(0.038)	(0.038)	(0.038)	(0.038)
60–100%	−0.024	−0.024	−0.025	−0.024	0.054	0.050	0.047	0.054
	(0.045)	(0.045)	(0.045)	(0.045)	(0.040)	(0.040)	(0.040)	(0.040)
Family cohesion (centered)	−0.053*	−0.054*	−0.053	−0.054*	−0.014	−0.017	−0.012	−0.014
	(0.030)	(0.030)	(0.030)	(0.030)	(0.027)	(0.027)	(0.027)	(0.027)
Parental warmth (centered)	−0.010	−0.010	−0.013	−0.011	0.016	0.014	0.007	0.015
	(0.022)	(0.022)	(0.022)	(0.022)	(0.020)	(0.020)	(0.020)	(0.020)
Parental monitoring (centered)	0.030	0.030	0.029	0.029	0.022	0.020	0.019	0.019
	(0.013)	(0.013)	(0.013)	(0.013)	(0.012)	(0.012)	(0.012)	(0.012)
Family cohesion*Change in heritage language use		−0.005				−0.059*		
		(0.029)				(0.025)		
Parental warmth*Change in heritage language use			−0.022				−0.076**	
			(0.021)				(0.019)	
Parental monitoring*Change in heritage language use				−0.025				−0.040
				(0.017)				(0.016)
No. of Obs.	1614	1614	1614	1614	1039	1039	1039	1039
Adjusted R-Squared	0.397	0.397	0.398	0.398	0.376	0.379	0.381	0.377

Standardized coefficients appear above standard errors in parenthesis

**p* < 0.05; ***p* < 0.01; ****p* < 0.001 (two-tailed tests)

^a^Reference: Southern and Other Europe (incl. Greece)

**Table 9:** OLS regression of internalizing and externalizing problems at wave 2 in the Netherlands

	Internalizing problems				Externalizing problems			
Variable	M1b	M2b	M3b	M4b	M5b	M6b	M7b	M8b
Internalizing/Externalizing problems at W1	0.556***	0.554***	0.556***	0.560***	0.561***	0.561***	0.561***	0.561***
	(0.028)	(0.028)	(0.028)	(0.028)	(0.028)	(0.028)	(0.028)	(0.028)
Change in heritage language use	−0.021	−0.020	−0.022	−0.023	−0.013	−0.013	−0.013	−0.014
	(0.019)	(0.019)	(0.019)	(0.019)	(0.014)	(0.014)	(0.014)	(0.014)
Heritage language use at W1	0.014	0.012	0.014	0.010	−0.041	−0.041	−0.041	−0.043
	(0.017)	(0.017)	(0.017)	(0.017)	(0.013)	(0.013)	(0.013)	(0.013)
Age	0.003	0.004	0.003	0.002	0.035	0.035	0.035	0.035
	(0.020)	(0.020)	(0.020)	(0.020)	(0.015)	(0.015)	(0.015)	(0.015)
Female	0.169***	0.170***	0.169***	0.164***	−0.124***	−0.124***	−0.124***	−0.126***
	(0.029)	(0.029)	(0.029)	(0.029)	(0.021)	(0.021)	(0.021)	(0.021)
*Immigrant generation* (ref = 1st generation)								
2nd generation	0.043	0.040	0.043	0.037	−0.007	−0.007	−0.007	−0.011
	(0.042)	(0.042)	(0.042)	(0.042)	(0.032)	(0.032)	(0.032)	(0.032)
3rd generation	0.106**	0.104*	0.106*	0.102*	0.031	0.032	0.031	0.029
	(0.056)	(0.056)	(0.056)	(0.056)	(0.043)	(0.043)	(0.043)	(0.043)
*Country of origin*^a^								
Turkey	−0.025	−0.025	−0.025	−0.022	−0.018	−0.018	−0.018	−0.017
	(0.055)	(0.055)	(0.055)	(0.055)	(0.042)	(0.042)	(0.042)	(0.042)
Morocco	−0.014	−0.015	−0.014	−0.010	0.006	0.006	0.006	0.008
	(0.052)	(0.052)	(0.052)	(0.051)	(0.039)	(0.039)	(0.039)	(0.039)
Suriname	−0.025	−0.025	−0.025	−0.025	−0.023	−0.023	−0.022	−0.022
	(0.050)	(0.050)	(0.050)	(0.050)	(0.038)	(0.038)	(0.038)	(0.038)
Indonesia	−0.016	−0.015	−0.016	−0.015	−0.006	−0.006	−0.006	−0.005
	(0.057)	(0.057)	(0.057)	(0.056)	(0.043)	(0.043)	(0.043)	(0.043)
Netherlands Antilles	−0.002	−0.003	−0.003	0.000	−0.010	−0.010	−0.010	−0.008
	(0.061)	(0.061)	(0.061)	(0.061)	(0.046)	(0.046)	(0.046)	(0.046)
Asia	0.056*	0.057*	0.056*	0.055*	−0.007	−0.007	−0.007	−0.007
	(0.055)	(0.055)	(0.055)	(0.054)	(0.041)	(0.041)	(0.041)	(0.041)
Africa	−0.040	−0.039	−0.040	−0.042	0.012	0.012	0.012	0.012
	(0.064)	(0.064)	(0.064)	(0.064)	(0.050)	(0.050)	(0.050)	(0.050)
Northern and Latin America	0.018	0.018	0.018	0.017	0.034	0.034	0.034	0.034
	(0.077)	(0.076)	(0.077)	(0.076)	(0.057)	(0.057)	(0.057)	(0.057)
Unknown country of origin	−0.038	−0.039	−0.038	−0.040	0.031	0.031	0.031	0.030
	(0.070)	(0.070)	(0.070)	(0.070)	(0.054)	(0.054)	(0.054)	(0.054)
*Stratum* (ref = 0–10%)								
10–30%	−0.023	−0.022	−0.023	−0.027	−0.000	−0.000	−0.000	−0.002
	(0.048)	(0.048)	(0.048)	(0.048)	(0.037)	(0.037)	(0.037)	(0.037)
30–60%	−0.003	−0.003	−0.003	−0.011	0.031	0.031	0.031	0.027
	(0.047)	(0.047)	(0.047)	(0.047)	(0.036)	(0.036)	(0.036)	(0.036)
60–100%	0.020	0.023	0.020	0.017	0.009	0.008	0.009	0.007
	(0.052)	(0.052)	(0.052)	(0.051)	(0.040)	(0.040)	(0.040)	(0.040)
Family cohesion (centered)	−0.013	−0.015	−0.013	−0.012	0.026	0.026	0.026	0.026
	(0.033)	(0.033)	(0.033)	(0.033)	(0.025)	(0.025)	(0.025)	(0.025)
Parental warmth (centered)	−0.047	−0.047	−0.047	−0.047	−0.054	−0.054	−0.054	−0.055
	(0.026)	(0.026)	(0.026)	(0.026)	(0.020)	(0.020)	(0.020)	(0.020)
Parental monitoring (centered)	0.006	0.006	0.006	0.004	−0.023	−0.023	−0.023	−0.023
	(0.016)	(0.016)	(0.016)	(0.016)	(0.012)	(0.012)	(0.012)	(0.012)
Family cohesion*Change in heritage language use		−0.043*				0.008		
		(0.033)				(0.026)		
Parental warmth*Change in heritage language use			−0.003				0.003	
			(0.024)				(0.018)	
Parental monitoring*Change in heritage language use				−0.063**				−0.034
				(0.019)				(0.014)
No. of Obs.	1203	1203	1203	1203	1129	1129	1129	1129
Adjusted R-Squared	0.431	0.432	0.430	0.434	0.367	0.366	0.366	0.367

Standardized coefficients appear above standard errors in parenthesis

**p* < 0.05; ***p* < 0.01; ****p* < 0.001 (two-tailed tests)

^a^Reference: Southern, Western, and Other Europe (incl. Germany)

**Table 10:** OLS regression of internalizing and externalizing problems at wave 2 in Sweden

	Internalizing problems				Externalizing problems			
Variable	M1c	M2c	M3c	M4c	M5c	M6c	M7c	M8c
Internalizing/Externalizing problems at W1	0.545***	0.544***	0.545***	0.546***	0.612***	0.614***	0.612***	0.611***
	(0.024)	(0.024)	(0.024)	(0.024)	(0.025)	(0.025)	(0.025)	(0.025)
Change in heritage language use	−0.016	−0.017	−0.015	−0.014	−0.008	−0.007	−0.008	−0.006
	(0.021)	(0.021)	(0.021)	(0.021)	(0.014)	(0.014)	(0.014)	(0.014)
Heritage language use at W1	−0.005	−0.004	−0.004	−0.002	0.003	0.003	0.003	0.005
	(0.015)	(0.015)	(0.015)	(0.015)	(0.010)	(0.010)	(0.010)	(0.010)
Age	−0.001	0.001	−0.001	−0.000	−0.027	−0.028	−0.027	−0.027
	(0.029)	(0.029)	(0.029)	(0.029)	(0.019)	(0.019)	(0.019)	(0.019)
Female	0.141***	0.141***	0.141***	0.140***	−0.025	−0.025	−0.025	−0.026
	(0.025)	(0.025)	(0.025)	(0.025)	(0.016)	(0.016)	(0.016)	(0.016)
*Immigrant generation* (ref = 1st generation)								
2nd generation	−0.024	−0.024	−0.024	−0.024	0.011	0.011	0.011	0.010
	(0.035)	(0.035)	(0.035)	(0.035)	(0.024)	(0.024)	(0.024)	(0.024)
3rd generation	−0.013	−0.012	−0.012	−0.010	0.007	0.007	0.007	0.008
	(0.054)	(0.054)	(0.054)	(0.054)	(0.035)	(0.035)	(0.035)	(0.035)
*Country of origin*^a^								
Former Yugoslavia	−0.035	−0.034	−0.034	−0.034	−0.001	−0.003	−0.001	−0.001
	(0.055)	(0.055)	(0.055)	(0.055)	(0.036)	(0.036)	(0.036)	(0.036)
Finland	0.011	0.012	0.011	0.012	0.028	0.026	0.028	0.030
	(0.052)	(0.052)	(0.052)	(0.052)	(0.032)	(0.032)	(0.032)	(0.032)
Iraq	−0.011	−0.009	−0.010	−0.010	−0.006	−0.009	−0.006	−0.005
	(0.066)	(0.066)	(0.066)	(0.066)	(0.045)	(0.045)	(0.045)	(0.045)
Turkey	0.004	0.006	0.005	0.004	0.008	0.006	0.008	0.008
	(0.064)	(0.064)	(0.064)	(0.064)	(0.042)	(0.042)	(0.042)	(0.042)
Somalia	−0.033	−0.033	−0.033	−0.034	−0.027	−0.028	−0.027	−0.027
	(0.087)	(0.087)	(0.087)	(0.087)	(0.058)	(0.058)	(0.058)	(0.058)
Middle East and Northern Africa (incl. Lebanon, Syria, Iran)	−0.014	−0.015	−0.014	−0.015	−0.020	−0.021	−0.020	−0.020
	(0.058)	(0.058)	(0.058)	(0.058)	(0.037)	(0.037)	(0.037)	(0.037)
Eastern and Other Africa	−0.020	−0.020	−0.019	−0.020	−0.046*	−0.045	−0.046*	−0.046*
	(0.076)	(0.076)	(0.076)	(0.076)	(0.051)	(0.051)	(0.051)	(0.051)
Asia	−0.012	−0.013	−0.013	−0.011	−0.017	−0.018	−0.017	−0.016
	(0.055)	(0.055)	(0.055)	(0.055)	(0.036)	(0.036)	(0.036)	(0.036)
Eastern and Other Europe (incl. Poland)	−0.009	−0.010	−0.009	−0.008	0.006	0.005	0.006	0.007
	(0.055)	(0.055)	(0.055)	(0.055)	(0.035)	(0.035)	(0.035)	(0.035)
Latin and Northern America	−0.018	−0.018	−0.018	−0.017	0.009	0.008	0.009	0.009
	(0.070)	(0.070)	(0.070)	(0.070)	(0.044)	(0.044)	(0.044)	(0.044)
Unknown country of origin	0.011	0.011	0.011	0.011	0.013	0.011	0.013	0.013
	(0.059)	(0.059)	(0.059)	(0.059)	(0.038)	(0.038)	(0.038)	(0.038)
*Stratum* (ref = 0–10%)								
10–30%	0.002	0.001	0.002	0.002	0.065	0.065	0.065	0.065
	(0.048)	(0.048)	(0.048)	(0.048)	(0.030)	(0.030)	(0.030)	(0.030)
30–60%	−0.005	−0.007	−0.005	−0.006	0.041	0.043	0.041	0.040
	(0.048)	(0.048)	(0.048)	(0.048)	(0.030)	(0.030)	(0.030)	(0.030)
60–100%	−0.011	−0.013	−0.011	−0.011	0.034	0.036	0.034	0.033
	(0.051)	(0.051)	(0.051)	(0.051)	(0.032)	(0.032)	(0.032)	(0.032)
Family cohesion (centered)	−0.018	−0.014	−0.020	−0.018	−0.035	−0.040	−0.035	−0.036
	(0.032)	(0.032)	(0.032)	(0.032)	(0.021)	(0.021)	(0.021)	(0.021)
Parental warmth (centered)	−0.042	−0.043	−0.039	−0.041	−0.029	−0.029	−0.029	−0.029
	(0.026)	(0.026)	(0.027)	(0.026)	(0.017)	(0.017)	(0.018)	(0.017)
Parental monitoring (centered)	0.005	0.005	0.006	0.002	−0.043*	−0.043*	−0.043*	−0.047*
	(0.014)	(0.014)	(0.014)	(0.014)	(0.009)	(0.009)	(0.009)	(0.009)
Family cohesion*Change in heritage language use		0.036				−0.036		
		(0.036)				(0.022)		
Parental warmth*Change in heritage language use			0.015				0.000	
			(0.028)				(0.017)	
Parental monitoring*Change in heritage language use				−0.041*				−0.030
				(0.020)				(0.013)
No. of Obs.	1794	1794	1794	1794	1486	1486	1486	1486
Adjusted R-Squared	0.385	0.386	0.385	0.387	0.415	0.416	0.415	0.416

Standardized coefficients appear above standard errors in parenthesis

**p* < 0.05; ***p* < 0.01; ****p* < 0.001 (two-tailed tests)

^a^Reference: Southern Europe, Germany, Denmark, and Norway

**Table 11:** OLS regression of internalizing and externalizing problems at wave 2 in the U.K.

	Internalizing problems				Externalizing problems			
Variable	M1d	M2d	M3d	M4d	M5d	M6d	M7d	M8d
Internalizing/Externalizing problems at W1	0.611***	0.611***	0.610***	0.612***	0.615***	0.618***	0.618***	0.614***
	(0.024)	(0.024)	(0.024)	(0.024)	(0.024)	(0.024)	(0.024)	(0.024)
Change in heritage language use	0.044*	0.044*	0.044*	0.034	−0.015	−0.005	−0.011	−0.005
	(0.023)	(0.023)	(0.022)	(0.023)	(0.016)	(0.016)	(0.016)	(0.017)
Heritage language use at W1	0.013	0.013	0.012	0.011	−0.009	−0.015	−0.013	−0.008
	(0.016)	(0.016)	(0.016)	(0.016)	(0.011)	(0.011)	(0.011)	(0.011)
Age	−0.019	−0.019	−0.021	−0.019	−0.011	−0.012	−0.011	−0.011
	(0.025)	(0.025)	(0.025)	(0.025)	(0.018)	(0.018)	(0.018)	(0.018)
Female	0.118***	0.118***	0.118***	0.118***	−0.016	−0.017	−0.014	−0.016
	(0.027)	(0.027)	(0.027)	(0.027)	(0.020)	(0.019)	(0.019)	(0.020)
*Immigrant generation* (ref = 1st generation)								
2nd generation	0.019	0.019	0.018	0.021	0.009	0.003	0.005	0.007
	(0.035)	(0.035)	(0.035)	(0.035)	(0.026)	(0.025)	(0.025)	(0.026)
3rd generation	0.044	0.044	0.042	0.045	0.038	0.030	0.032	0.036
	(0.047)	(0.047)	(0.047)	(0.047)	(0.034)	(0.034)	(0.034)	(0.034)
*Country of origin*^a^								
Pakistan	−0.033	−0.033	−0.034	−0.031	−0.052	−0.054	−0.056	−0.053
	(0.052)	(0.052)	(0.052)	(0.052)	(0.038)	(0.037)	(0.037)	(0.038)
Asia (incl. Bangladesh)	−0.055*	−0.055*	−0.057*	−0.055*	−0.052	−0.056	−0.058	−0.052
	(0.053)	(0.053)	(0.053)	(0.053)	(0.038)	(0.038)	(0.038)	(0.038)
India	−0.041	−0.041	−0.042	−0.042	−0.027	−0.031	−0.029	−0.027
	(0.051)	(0.051)	(0.051)	(0.051)	(0.036)	(0.036)	(0.036)	(0.036)
Eastern and Western Africa (incl. Nigeria)	−0.064*	−0.064*	−0.064*	−0.063*	−0.040	−0.040	−0.038	−0.041
	(0.053)	(0.053)	(0.053)	(0.053)	(0.039)	(0.038)	(0.038)	(0.039)
Latin America and the Caribbean (incl. Jamaica)	−0.032	−0.032	−0.032	−0.032	−0.032	−0.035	−0.034	−0.032
	(0.057)	(0.057)	(0.057)	(0.057)	(0.040)	(0.040)	(0.040)	(0.040)
Other Africa	−0.026	−0.026	−0.026	−0.026	−0.023	−0.026	−0.026	−0.022
	(0.073)	(0.073)	(0.073)	(0.073)	(0.054)	(0.054)	(0.054)	(0.054)
Eastern Europe	−0.015	−0.015	−0.017	−0.013	−0.020	−0.021	−0.024	−0.021
	(0.093)	(0.093)	(0.093)	(0.093)	(0.072)	(0.071)	(0.071)	(0.072)
Northern America and Oceania	−0.017	−0.017	−0.018	−0.018	−0.009	−0.010	−0.010	−0.009
	(0.090)	(0.090)	(0.090)	(0.090)	(0.067)	(0.066)	(0.066)	(0.067)
Unknown country of origin	−0.032	−0.032	−0.033	−0.032	−0.014	−0.016	−0.016	−0.014
	(0.048)	(0.048)	(0.048)	(0.048)	(0.035)	(0.034)	(0.034)	(0.035)
*Stratum* (ref = 0–10%)								
10–30%	−0.047	−0.047	−0.046	−0.046	−0.156***	−0.155***	−0.156***	−0.157***
	(0.063)	(0.063)	(0.063)	(0.063)	(0.046)	(0.045)	(0.046)	(0.046)
30–60%	−0.054	−0.054	−0.054	−0.054	−0.169***	−0.166***	−0.169***	−0.168***
	(0.065)	(0.065)	(0.065)	(0.065)	(0.047)	(0.047)	(0.047)	(0.047)
60–100%	−0.091	−0.090	−0.089	−0.090	−0.175**	−0.165**	−0.169**	−0.174**
	(0.065)	(0.065)	(0.065)	(0.065)	(0.047)	(0.047)	(0.047)	(0.047)
Independent schools	−0.018	−0.018	−0.018	−0.019	−0.076*	−0.075*	−0.076*	−0.076*
	(0.072)	(0.072)	(0.072)	(0.072)	(0.051)	(0.051)	(0.051)	(0.051)
Family cohesion (centered)	−0.100***	−0.100***	−0.099***	−0.101***	−0.064*	−0.072*	−0.060	−0.064*
	(0.030)	(0.030)	(0.030)	(0.030)	(0.022)	(0.021)	(0.021)	(0.022)
Parental warmth (centered)	0.075**	0.075**	0.072*	0.076**	0.041	0.043	0.031	0.040
	(0.025)	(0.025)	(0.025)	(0.025)	(0.018)	(0.018)	(0.018)	(0.018)
Parental monitoring (centered)	0.031	0.031	0.031	0.034	−0.043	−0.043	−0.045*	−0.045*
	(0.014)	(0.014)	(0.014)	(0.014)	(0.011)	(0.011)	(0.011)	(0.011)
Family cohesion*Change in heritage language use		−0.001				−0.080***		
		(0.033)				(0.023)		
Parental warmth*Change in heritage language use			−0.028				−0.077***	
			(0.030)				(0.021)	
Parental monitoring*Change in heritage language use				0.043*				−0.034
				(0.025)				(0.017)
No. of Obs.	1359	1359	1359	1359	1201	1201	1201	1201
Adjusted R-Squared	0.449	0.448	0.449	0.450	0.421	0.427	0.426	0.421

Standardized coefficients appear above standard errors in parenthesis

**p* < 0.05; ***p* < 0.01; ****p* < 0.001 (two-tailed tests)

^a^Reference: Southern and Other Europe (incl. Ireland)

## Footnotes

1 Nevertheless, their results suggest that support from friends and teachers moderated the association of ethnic culture competence and mental health, whereas family support moderated the association of host culture competence and mental health.

2 Nonresponse rates that include those who did not answer either of the wave 1 and 2 survey question on internalizing/externalizing problems range from 3.44 to 15.52% for internalizing problems (15.52% in Germany, 3.44% in the Netherlands, 7.23% in Sweden, 13.76% in the U.K.), which are lower than nonresponse rates for externalizing problems that range from 9.21 to 42.54% (42.54% in Germany, 9.21% in the Netherlands, 24.51% in Sweden, 24.37% in the U.K.). Germany has very high nonresponse rates for externalizing problems, which is mainly due to the exclusion of several questions related to delinquent behaviors in one federal state of Germany in Wave 1 and problem behaviors in some versions of the Wave 2 German questionnaire (see CILS4EU codebooks).

3 The original filtering question about language use at home asks “Is there a language other than <survey country language> spoken at your home?” and followed by a question asking “In this (second) language, how often do you talk to your family?”. We follow prior studies (e.g., Jasini et al., 2019; Kogan & Weißmann, 2019) and assume that another language spoken at home is one’s heritage language.

4 Some cases with incomplete information were included in the 2nd generation category (*n* = 22 in internalizing samples, *n* = 14 in externalizing samples): parents foreign-born but no information on child or child native born and grandparents foreign-born but no information on parents.

5 Also, there is possibility that family relations and heritage language use could be bidirectionally associated, suggesting the moderating effect of heritage language use on the relationship between family relations and behavioral and mental health problems. However, as noted, the current data do not have family relations measures at wave 2. Future research with longitudinal data could test this possibility.
